# Tooth Wear in a Sample of Community-Dwelling Elderly Greeks

**DOI:** 10.3290/j.ohpd.a43348

**Published:** 2020-02-12

**Authors:** Eleftherios G Kaklamanos, Georgios Menexes, Miltiadis A Makrygiannakis, Vassiliki Topitsoglou, Sotirios Kalfas

**Affiliations:** a Associate Professor, Hamdan Bin Mohammed College of Dental Medicine, Mohammed Bin Rashid University of Medicine and Health Sciences, Dubai, United Arab Emirates. Conceived the study and contributed to its design, contributed to data collection, contributed to data analysis and interpretation, preparation and revision of the manuscript.; b Assistant Professor, Laboratory of Agronomy, School of Agriculture, Faculty of Agriculture, Forestry and Natural Environment, Aristotle University of Thessaloniki, Thessaloniki, Greece. Contributed to study design, data analysis and interpretation, preparation and revision of manuscript.; c Orthodontic Specialist, Private practice, Athens, Greece. Contributed to data analysis and interpretation, preparation and revision of manuscript.; d Professor, Department of Preventive Dentistry Periodontology and Implant Biology, School of Dentistry, Faculty of Health Sciences, Aristotle University of Thessaloniki, Thessaloniki, Greece. Conceived the study, contributed to study design, data collection, data analysis and interpretation, preparation and revision of manuscript.; e Professor, Department of Preventive Dentistry Periodontology and Implant Biology, School of Dentistry, Faculty of Health Sciences, Aristotle University of Thessaloniki, Thessaloniki, Greece. Conceived the study and contributed to its design, contributed to data collection, contributed to data analysis and interpretation, preparation and revision of manuscript.

**Keywords:** tooth surface loss, prevalence, extent, elderly, tooth wear index

## Abstract

**Purpose:**

Increased wear of teeth may constitute a major problem in the future for the elderly. The aim of the present study was to investigate tooth wear in a sample of elderly Greeks and explore the presence and extent of severe occlusal/incisal wear in relation to the parameters of age, gender and remaining teeth.

**Materials and Methods:**

A convenience sample of 70 dentate elderly (60–92 years old) was examined. Tooth wear was assessed using a modification of the tooth wear index. The presence of severe occlusal/incisal wear was explored using the multiple correspondence analysis (MCA) and *x*^2^ tests and the extent (% of surfaces) with analysis of variance (ANOVA).

**Results:**

Increased prevalence of severe wear was observed in the occlusal/incisal and, to a lesser extent, in the cervical surfaces of the examined teeth. Seventy per cent (70%) of the participants had at least one severely worn tooth surface. Advanced age seemed to be associated with severe occlusal/incisal wear (>70 years: 79.4%; 60–70 years: 52.8%; *x*^2^ test, p = 0.024). The mean (± SD) percentage of severely worn teeth and surfaces was 34.2 ± 32.6% and 9.6 ± 9.6%, respectively. ANOVA showed that males and those with less than 20 remaining teeth exhibited more severely worn occlusal/incisal surfaces (p = 0.031 and p = 0.000, respectively).

**Conclusions:**

The presence and the extent of severe wear in the sample of the examined elderly is high compared to elderly populations in other countries. Severe wear was more prevalent with advanced age and more extensive in the occlusal/incisal surfaces in males and those having less than 20 remaining teeth.

Tooth wear is an irreversible and cumulative phenomenon related to aesthetic and functional disorders. Although it should not be considered as a part of the ageing process, a general tendency towards increasing wear with age has been well established.^27^ With increasing life expectancy, the population is ageing and a growing number of elderly people are retaining more of their natural dentition. The increased loss of dental tissue as a result of wear may constitute a major problem in the future.^19,21^

Apart from age-related changes, variations in the rates of tooth wear in the general population have also been associated with other parameters. Most studies have suggested that men exhibit increased wear compared to women.^27^ In addition, the increase in the number of teeth lost has been associated with more severe wear of those remaining, although this association is not always clinically or statistically significant.^29^ Moreover, underlying systemic predisposing factors, dietary habits, as well as social and environmental difficulties may also have an impact on the wear of teeth.^18,28,29^ However, the in-depth understanding of the parameters affecting interindividual variations in the manifestations of the tooth wear process is still limited.

Elderly people’s circumstances may vary considerably and under certain conditions they may be more at risk in terms of a deterioration in oral health.^9^ Given the growing concern about maintaining the level of health in the growing population of elderly people, and their oral health in particular, the complexity of treatment and the potential impacts of advanced tooth wear on the Oral Health-related Quality of Life, additional epidemiological information about the prevalence of tooth wear has become a matter of interest.^12,15,29^ The available data is relatively sparse and indicate considerable variation in the proportions of the elderly population presenting with teeth worn to such a degree that function or appearance may be impaired, or in the extent of advanced wear in their dentition.^2,7,24,18,28^

The aim of this paper was to investigate tooth wear in a sample of community-dwelling elderly Greeks and explore the presence and extent of severe occlusal/incisal wear in relation to the parameters of age, gender and remaining teeth.

## Materials and Methods

### Participants

In total, a convenience sample of 70 dentate elderly people were examined. All elderly living independently and attending an elderly people’s day centre (EPDC) in one of five different suburban areas of Thessaloniki, Greece were invited to participate. The age of the participants ranged from 60 to 92 years. Approval was obtained by the Research and Ethics Committee, Aristotle University of Thessaloniki and informed consent was obtained from the participants.

### Clinical Examination

The clinical examinations were performed by one investigator (EGK), using plane mirrors and blunt explorers under natural light conditions in a specially designated office of the EPDC. Disposable latex gloves and single-use sterilised instruments were used for each participant. The clinical examination involved an assessment of the number of remaining teeth (excluding root remnants), as well as observation of the presence of complete or partial dentures. The findings of the clinical examination were recorded on precoded forms.

Tooth wear was assessed using the modified tooth wear index (mTWI).^3^ The index has been used to develop maximum scores of expected wear for various age groups, depending on tooth surface and position.^23^ Occlusal/incisal wear scored 3 to 5 in the mTWI was regarded as affecting function or aesthetics for the age groups under consideration and is hereafter mentioned as severe wear. The extent of severely worn surfaces was expressed as a percentage of the total or occlusal/incisal surfaces scored.

To assess the intraexaminer reliability, 20 of the elderly participants were re-examined after approximately 3 weeks and a kappa score was calculated.

### Statistical Methods

The statistical significance of the differences between males and females regarding general characteristics was analysed with the Mann–Whitney test or the *x*^2^ test.

The presence of severe occlusal/incisal wear in relation to the parameters of age, gender and the remaining teeth distribution was explored with multiple correspondence analysis (MCA) and investigated with the *x*^2^ test. The extent of severe occlusal/incisal wear (% of surfaces) in relation to the effects of gender, age and remaining teeth distribution was investigated with analysis of variance (ANOVA). In order to maximise the adjustment of the percentage distribution to the normal distribution, the transformation *T* = *2arcsin*($\sqrt{X}$) was applied, where *X* is the percentage value.

The patterns of teeth exhibiting severe occlusal/incisal wear in each quadrant were identified by creating a new variable *v_Tqn_* for each tooth, where *q = 1,... 4* represents the quadrant and *n = 1,... 8* the tooth number in the quadrant. If a tooth was severely worn, the variable *v_Tqn_* took the value 1. Otherwise, the variable *v_Tqn_* took the value 0. The pattern of severely worn teeth in each quadrant was created according to the relationship *P(T_q1_ + T_q2_ + T_q2_) = 10^7^v_Tq1_ + 10^6^v_Tq2_ +...+ 10^0^v_Tq8_*, where *q = 1,... 4* represents the quadrant. The patterns obtained were used to create frequency tables for each quadrant.

The software SPSS Statistics version 22 (IBM, Chicago, IL, USA) was used for the analyses. In every non-parametric test (*x*^2^ tests and Mann–Whitney) the observed statistical significance level (p value) was computed either with the Fisher’s exact test or using the Monte Carlo simulation method. The statistical tests differences were considered statistically significant when a p value of 0.05 or less was found.

## Results

The kappa score for intraexaminer reliability was calculated as 0.853, indicating a very close intraexaminer agreement. The general characteristics of the participants are presented on Table 1. No statistically significant association was found between gender and the remaining teeth distribution (*x*^2^ test; p = 0.641).

**Table 1:** Characteristics of the participants. The statistical significance of the differences between males and females was analysed with the Mann–Whitney test (indicated by an asterisk) or by the *x*^2^ test

Characteristic	Elderly aged 60–70 years	Elderly aged over 70 years	Statistical significance	Total
**Gender distribution [no. (%)]**				
Males Females Remaining teeth [*x* *_* ± SD]*	15 (42%) 21 (58%) 18 ± 7	21 (62%) 13 (38%) 18 ± 6	0.102 0.777	36 (51%) 34 (49%) 18 ± 6
**Remaining teeth distribution [no. (%)]**				
Less than 20 teeth 20 or more teeth	19 (53%) 17 (47%)	18 (53%) 16 (47%)	1.000	37 (53%) 33 (47%)
**Wearing of dentures [no. (%)]**				
Non-denture wearers Denture wearers Partial denture(s) Complete & partial dentures Complete denture & teeth	20 (56%) 16 (44%) 12 (33%) 4 (11%) 0 (0%)	19 (56%) 15 (44%) 11 (32%) 2 (6%) 2 (6%)	0.606	39 (56%) 23 (33%) 6 (8%) 2 (3%)

**Fig 1:**
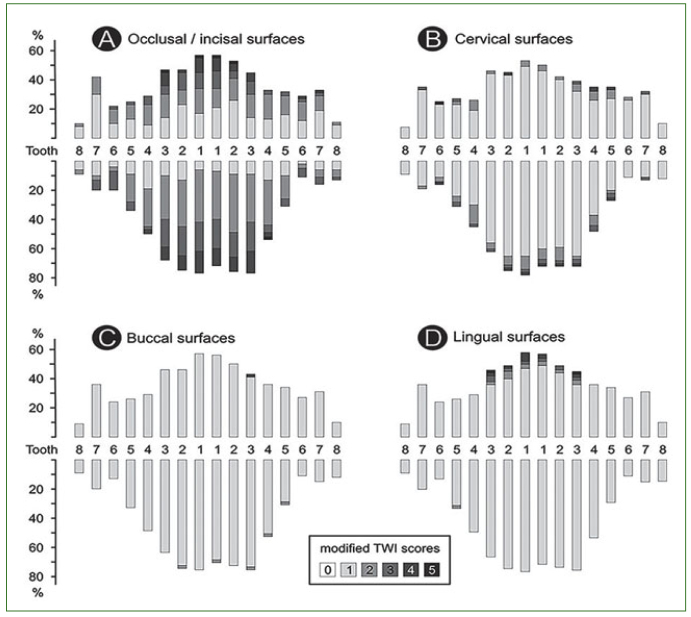
Distribution of the modified tooth wear index (mTWI) scores in the dentition of the elderly.

The number of tooth surfaces scored for wear and the distribution of wear in the dentition of the 70 elderlies are presented in Table 2 and Figure 1, respectively. An increased proportion of severe wear was observed in the occlusal/incisal and to a lesser extent in the cervical surfaces of the examined teeth. With the exception of the lingual surfaces of the maxillary anterior teeth, the buccal and lingual surfaces did not exhibit severe wear overall. Seventy per cent (70%) of the elderly participants exhibited at least one severely worn surface. The mean (± SD) percentage of severely worn teeth and surfaces in the dentition was 34.2% ± 32.6 and 9.6% ± 9.6, respectively.

**Table 2:** Number of tooth surfaces scored for wear

	8	7	6	5	4	3	2	1	Tooth surface	1	2	3	4	5	6	7	8
**Maxilla**	6	24	17	18	18	32	31	37	**Cervical**	35	29	26	24	24	19	22	7
	6	25	17	18	20	32	32	40	**Buccal**	39	35	30	25	24	19	22	7
	6	25	17	17	20	32	32	40	**Occlusal/incisal**	39	35	31	23	22	19	22	7
	6	25	17	18	20	31	33	40	**Lingual**	39	34	30	25	24	19	22	7
**Mandible**	6	14	9	23	31	46	52	53	**Lingual**	50	51	52	37	20	8	10	8
	6	14	9	23	34	47	52	53	**Occlusal/incisal**	50	51	53	37	21	7	10	8
	6	14	9	23	34	45	52	53	**Buccal**	50	51	53	37	21	8	10	8
	6	13	9	22	31	43	51	53	**Cervical**	50	49	49	34	18	8	9	8

### Presence of Severe Occlusal/Incisal Wear

**Fig 2:**
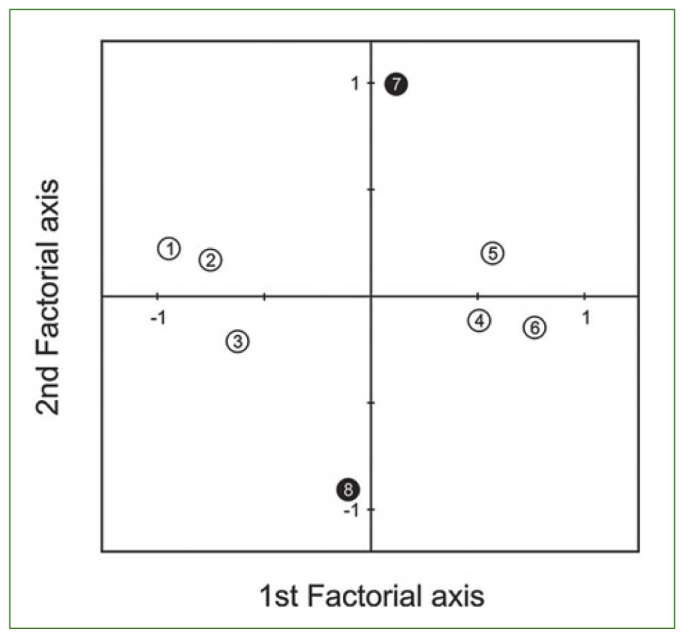
The factorial plane 1 × 2 showing the prevailing trends, as derived by MCA. The first axis is mainly constructed by points 1–6 (open circles), while the second is mainly constructed by points 7 and 8 (filled circles). Point 1: female elderly participants; point 2: elderly aged 60–70 years old; point 3: absence of severe occlusal/incisal wear; point 4: presence of severe occlusal/incisal wear; point 5: male elderly participants; point 6: elderly aged over 70 years; point 7: elderly having less than 20 remaining teeth; point 8: elderly having 20 remaining teeth or more.

MCA revealed two statistically significant factorial axes that explained almost 61% of the total variance regarding the presence of severely worn occlusal/incisal surfaces (Fig 2). The first axis showed that the parameters of age (discrimination measure (DM) = 0.56) and gender (DM = 0.36) were related to the presence of advanced wear (DM = 0.49) and differentiated elderly females, aged 60–70 years, without severe wear (left side of the axis), from males over 70 years with severe wear (right side of the axis). The second factorial axis was composed almost exclusively of the parameter relevant to the number of remaining teeth distribution (DM = 0.92) and differentiated persons having 20 or more remaining teeth (positive side of the axis) from those with less than 20. The number of remaining teeth did not seem to be significantly connected to the presence of severe wear or the parameters of age and gender. Further testing with a series of *x*^2^ tests showed that the persons over 70 exhibited more severe wear compared to those 70 and younger (79.4% vs 52.8%; p = 0.024), especially in the subgroup with less than 20 remaining teeth (83.4% vs 47.4%; p = 0.001)

### Extent of Severe Occlusal/Incisal Wear

ANOVA showed that the extent of severe wear was greater in males (mean ± SD: 54.4 ± 30.3% vs 41.2 ± 29.0% in females; F(1:38) = 5.046; p = 0.031) and in persons with less than 20 remaining teeth (mean ± SD: 32.4 ± 22.0% vs 63.6 ± 29.2% in persons with less than 20 remaining teeth; F(1:38) = 22.983; p = 0.000).

### Patterns of Severe Occlusal/Incisal Wear

**Fig 3:**
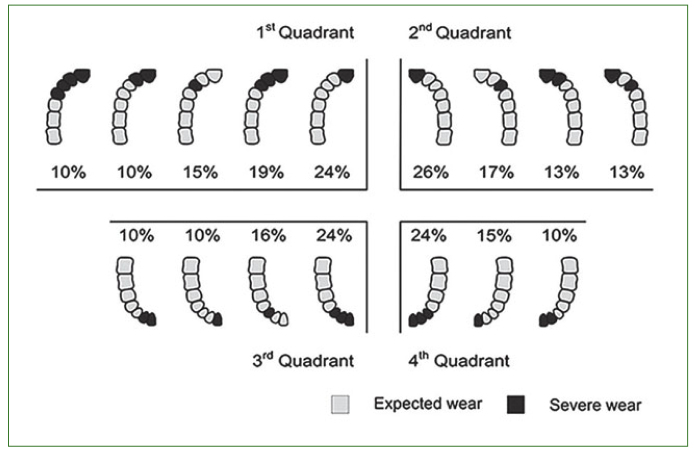
Frequent patterns of severely worn occlusal/incisal surfaces.

The more frequent patterns of severely worn occlusal/incisal surfaces are presented in Fig 3. Severe wear involved mainly the incisal surfaces of the anterior teeth.

## Discussion

Based on data analysis, the presence and the extent of severe wear was high in the sample of the examined elderly. Moreover, severe wear in the occlusal/incisal surfaces was more prevalent in elderly more than 70 years old, and extensive in males and the elderly with less than 20 remaining teeth.

The published data on the presence and extent of tooth wear in the general elderly population people are relatively sparse, as the majority of the work published concerns other age cohorts, particular varieties of tooth wear or specific settings.^1,3,5,11,14,20,22^ The available data involves investigations from Sweden,^7^ the United Kingdom,^3,10,24^ Ireland,^2^ the Netherlands,^28^ and the United States^18^ that have used the TWI^23^ or modifications,^2,4^ the O’Brien index,^17^ the Lobbezoo and Naeije index,^13^ or grading scales produced by the authors.^7^ In the present investigation, the severity of tooth wear was assessed using the mTWI.^3^ This modification grades the extent of surface area lost by wear in the horizontal plane, but additionally includes a criterion for assessing vertical crown loss, which has been considered important for further differentiation of the ongoing progress of tooth substance loss in elderly people. Moreover, the index has been used to develop maximum scores of expected wear for various age groups depending on tooth surface and position.^23^

Although the variability of the grading scales used renders unequivocal comparison challenging,^14^ it seems that the sample of investigated elderly Greeks included a considerable subset of individuals demonstrating teeth with advanced wear level (70%) compared to elderly populations in other countries (reported range: 12–50%).^2,10,18,24,28^ Moreover, the examined individuals exhibited a high percentage (34.2%) of advanced worn teeth (reported range: 0.8–19.5%).^2,7,10^ Added to this, a general tendency of increase in tooth substance loss was observed on the occlusal/incisal, cervical, as well as the lingual surfaces of the anterior teeth rather than among the posterior teeth, similarly to other reports.^3^

In the context of increased tooth longevity, tooth wear may constitute an increasing future problem for populations with characteristics similar to those of the examined elderly. Bearing in mind the potential impacts on oral health-related quality of life and the complexity of treatment,^12,15^ this finding may suggest increased treatment needs, although the decision to provide treatment depends on a wide range of parameters including age, gender, socioeconomic factors and desire for care.^3^ In any case, increasingly complex restorations may be needed, requiring time and the relevant skills to perform and maintain.

MCA and *x*^2^ tests revealed that the presence of severe occlusal/incisal wear seems to be related to males and people over 70 years. In addition, ANOVA showed that the percentage of severely worn occlusal/incisal surfaces is increased in males and those with less than 20 remaining teeth. The observation that tooth wear increases with age is well established.^3,7,16,18,24,25,27-29^ Since tooth wear constitutes a non-reversible process, such findings are to be expected. Moreover, a general tendency for men to exhibit increased wear compared to women has been observed in the published literature.^2,3,5,7,18,28^ Dietetic factors,^6^ as well as the forces arising from orofacial muscle function^26^ have been implicated in the attempts to explain these observations. Finally, the increase in the number of teeth lost has been associated with more severe wear in the remaining dentition, although this association has not been unequivocally established.^8,29^

## Conclusions

Based on the retrieved data, the presence and the extent of severe wear was high in the sample of the examined elderly compared to elderly populations in other countries. Moreover, severe wear became more prevalent with advancing age and was more extensive in the occlusal/incisal surfaces in males and those with less than 20 remaining teeth. In the context of increased tooth longevity, tooth wear may become an increasing problem in the future for the rapidly ageing population, implying more and more complex restorations, requiring time and skills to perform and maintain. Further studies are needed to investigate tooth wear in older Greeks and clarify the determinants of inter-individual variations.
